# Treatment with Geranylgeranylacetone Induces Heat Shock Protein 70 and Attenuates Neonatal Hyperoxic Lung Injury in a Model of Bronchopulmonary Dysplasia

**DOI:** 10.1007/s00408-017-0007-4

**Published:** 2017-04-26

**Authors:** Shuko Tokuriki, Aiko Igarashi, Takashi Okuno, Genrei Ohta, Hironobu Naiki, Yusei Ohshima

**Affiliations:** 1 Department of Pediatrics, Faculty of Medical Sciences, University of Fukui, 23-3 Shimoaizuki, Matsuoka, Eiheiji-cho, Yoshida-gun, Fukui, 910-1193 Japan; 2 Department of Pathological Sciences, Faculty of Medical Sciences, University of Fukui, 23-3 Shimoaizuki, Matsuoka, Eiheiji-cho, Yoshida-gun, Fukui, 910-1193 Japan

**Keywords:** Bronchopulmonary dysplasia, Geranylgeranylacetone, Hyperoxia, Alveolarization

## Abstract

**Purpose:**

Bronchopulmonary dysplasia (BPD) is a respiratory complication characterized by abnormal alveolar development in premature infants. Geranylgeranylacetone (GGA) can induce heat shock protein (HSP) 70, which has cytoprotective effects against various stressors. Here, we investigated whether GGA protected neonatal lungs from hyperoxic stress in a murine BPD model, and measured the serum HSP70 levels in preterm humans treated with oxygen.

**Methods:**

Newborn mice were exposed to >90% oxygen and administered GGA or vehicle alone orally on days 1, 2, and 3 of life. At 2 days of age, HSP70 expression in the lung was determined by western blotting. At 8 days of age, the lungs were processed for histological analysis. Radial alveolar count (RAC) and mean linear intercept (MLI) were measured as parameters of alveolarization. Apoptosis was evaluated by the terminal deoxynucleotidyl transferase-mediated dUTP nick end labeling (TUNEL) method and cleaved caspase-3 immunohistochemistry. Serum HSP70 levels in preterm humans treated with oxygen were measured by enzyme-linked immunosorbent assay.

**Results:**

GGA administration enhanced the HSP70 expression to two-fold compared with normoxia-exposed and vehicle-treated mice. Hyperoxia reduced HSP70 expression, whereas GGA abrogated the effects. Hyperoxia-exposed mice exhibited more apoptotic cells in lung parenchyma and a more simplified alveolar structure with less RAC and larger MLI than normoxia-exposed mice. GGA suppressed the increase in apoptotic cells and the structural changes of the lungs induced by hyperoxia. Serum HSP70 levels of preterm human infants gradually decreased with age.

**Conclusions:**

GGA may attenuate hyperoxic injury in neonatal lungs and thereby may prevent the development of BPD.

## Introduction

Bronchopulmonary dysplasia (BPD) is a major respiratory complication in premature infants [1]. The etiology of BPD is multifactorial, and includes oxygen toxicity, ventilator-induced pulmonary injury, and antenatal and/or postnatal inflammation [2, 3]. BPD is characterized by poor alveolarization and vascularization in the developing lungs of immature infants [4]. Currently, effective postnatal therapies are limited and new therapeutic interventions to prevent or treat BPD are required.

Heat shock proteins (HSPs) constitute a family of intracellular proteins activated by a variety of stressors, including ischemia and inflammation. HSPs assist in delivering target proteins to the ubiquitin–proteasome system for degradation together with cochaperone molecules [5, 6]. Among them, HSP70 has a cytoprotective function via anti-apoptotic and anti-inflammatory effects in vitro and in vivo. Increased expression of HSP70 attenuates hyperoxia-induced lipid peroxidation and ATP depletion in human respiratory epithelial cells in vitro [7]. HSP70-deficient mice exhibit increased mortality under hyperoxia, and adenoviral delivery of HSP70 to the lungs effectively rescues both HSP70-deficient and wild-type mice [8]. The induction of HSP70 is a potential therapeutic target against several stressor-induced lung injuries.

Geranylgeranylacetone (GGA), an acyclic polyisoprenoid widely used for ulcer therapy, induces HSP70 expression [9, 10]. GGA exhibits cytoprotective and anti-inflammatory effects in bleomycin-induced lung fibrosis mouse models [11, 12] and reduces hydrogen peroxide-induced apoptotic cell death by enhancing HSP production [13]. GGA prevents oxidative stress in liver, brain, kidney, and retina. However, whether GGA administration can prevent hyperoxia-induced neonatal lung injury remains unclear. Thus, we examined the effects of GGA on alveolarization of neonatal lungs exposed to hyperoxia in a murine BPD model.

## Methods

### Animals and Study Design

C57BL/6J mice were obtained from Charles River Japan (Shizuoka, Japan). Neonatal pups were randomly divided into four groups based on hyperoxia exposure and GGA treatment. The pups were kept in room air (normoxia) unless exposed to hyperoxia with an oxygen concentration of >90% in a 46.7-liter Plexiglas exposure chamber (Fukui, Japan) from 1 to 3 days of age. Maternal mice were swapped every 24 h between normoxia and hyperoxia groups. Each mother was given four to eight pups. The pups were treated with 500 mg/kg/day of GGA (Tokyo Chemical Industry Co., Tokyo, Japan) or 10 ml/kg of vehicle alone (5% arabic gum and 0.6% Tween 80) during the 3 days of hyperoxia exposure. As the body weight of 1–3-day-old pups was only 1.0–2.0 g, we administered 10 μl of 5% GGA solution (50 mg/ml) per 1 g of body weight. All pups were anesthetized by isoflurane inhalation and orally administered GGA or vehicle using the outer cylinders of 24-gauge indwelling needles. In order to confirm reliable administration of GGA or vehicle into their stomach, orally administered solution was stained blue using a food-coloring agent. GGA emulsified in vehicle was freshly prepared and then administered to the mice once a day. At 2 and 8 days of age, pups were sacrificed to obtain lung tissue. The study protocol was approved by the Experimental Animal Ethics Committee at Fukui University (Permit No. 26129).

### Study Population of Human Neonates and Measurement of Serum HSP70

Between March 2012 and April 2015, 347 infants were admitted to the Neonatal Intensive Care Unit, Growing Care Unit, or Nursery at the University of Fukui Hospital. Twenty-six infants were excluded due to death, chromosomal abnormality, metabolic diseases, severe intraventricular hemorrhage, and surgical diseases or a hospital transfer before 1 month of age. Fifty-seven of the remaining 321 infants, of which serum samples had been obtained between postnatal days 0 and 3, were enrolled in this study. Of these, serum samples of 10 infants born at <32 weeks of gestational age had also been obtained between postnatal days 28 and 30. Demographic and clinical data regarding the infants’ perinatal and postnatal courses were extracted from the medical records. The independent variables were gestational age, birth weight, sex, prenatal steroid use, Apgar scores at 1 and 5 min, surfactant use, the presence of histologically determined chorioamnionitis (Stage 2 or 3) [14], and BPD, defined as oxygen dependence at 36 weeks of postmenstrual age. The samples were frozen at −20 °C until they were analyzed. HSP70 levels were measured in duplicate using enzyme-linked immunosorbent assay kits (Enzo Life Sciences Inc., NY, USA). The study was approved by the Institutional Ethics Committee at the University of Fukui.

### Western Blotting Analysis of HSP70 Expression

Murine lung tissues resected at 2 days of age were homogenized in ice-cold tissue lysis buffer consisting of 10 mM Tris base-pH 7.4, 0.1% sodium dodecyl sulfate (SDS), 150 mM NaCl, 1 mM ethylenediaminetetraacetic acid (EDTA), 1% octylphenoxypolyethoxyethanol, 0.1% sodium deoxycholate, and protease inhibitor. The total extracted cell proteins were electrophoresed by SDS-PAGE (20 μg protein/lane). Proteins were transferred to a polyvinylidene difluoride membrane (Bio-Rad Laboratories Inc., CA) and immunoblotted with polyclonal rabbit anti-HSP70 antibody (Enzo Life Sciences, Inc., NY, USA) or anti-β-actin antibody (Cell Signaling Technology, Danvers, USA). Blots were then incubated with appropriate HRP-conjugated secondary antibodies and bands were visualized using ImmunoStar^®^ (Wako Pure Chemical Industries, Ltd., Osaka, Japan). The amount of protein on the membrane was quantified by a chemiluminescence imaging analyzer (ImageQuant LAS 4000mini, GE Healthcare, Tokyo, Japan) and the expression levels were recorded as ratios to the level of β-actin.

### Tissue Preparation and Morphological Assessment of the Lung

After euthanasia, the pulmonary artery was perfused with saline. The right lung was embedded in optimal cutting temperature (OCT) compound (Tissue-Tek^®^; Sakura, Japan) and frozen for immunohistochemistry. The left lung was inflated with 10% formaldehyde at 20 cm H_2_O pressure by clamping the trachea. Sections from the formalin-fixed, paraffin-embedded blocks were stained with hematoxylin and eosin for morphological evaluation. At least 10 images of the lung tissue were randomly recorded in a blinded fashion with a charge-coupled device camera system (OLYMPUS FX380; Olympus Co., Tokyo, Japan). Alveolarization was assessed by measuring radical alveolar counts (RAC) and mean linear intercept (MLI), as described previously [15, 16].

### Evaluation of Apoptosis in Murine Lung Tissue

A terminal deoxynucleotidyl transferase-mediated dUTP nick end labeling (TUNEL) assay was performed using the In Situ Cell Death Detection Kit (Roche Diagnostics GmbH, Mannheim, Germany) according to the manufacturer’s instructions. Fluorescence microscope images were used to calculate the area stained with TUNEL using Metamorph software (Molecular Devices, Sunnyvale, CA, USA).

To evaluate cleaved caspase-3, 10-μm frozen lung tissue sections were fixed in 4% paraformaldehyde solution for 20 min at room temperature, permeabilized with 0.2% Triton^®^X-100 in phosphate-buffered saline (PBS) for 2 min on ice, and incubated with blocking buffer (Blocking One Histo^®^; Nacalai Tesque, Inc., Kyoto, Japan) for 10 min at room temperature, followed by incubation with anti-active caspase-3 pAb (Promega Co., Madison, WI, USA) (1:100) overnight at 4 °C, and then Alexa Fluor 488 goat anti-rabbit immunoglobulin G (1:5000) for 60 min at room temperature. Samples were mounted with Prolong^®^ (Thermo Fisher Scientific, Waltham, MA, USA) and inspected under a fluorescence microscope. The number of cleaved caspase-3-positive cells in the whole area of a section was counted and expressed as per the total area calculated by Metamorph software.

### Statistical Analysis

Murine data are presented as mean ± standard error of the mean (SEM), and were analyzed using one-way analysis of variance (ANOVA) or Student’s *t* test. Human data are presented as medians and ranges, and were analyzed using the Mann–Whitney *U* and Wilcoxon signed-rank tests. Correlations were evaluated using the non-parametric Spearman’s rho test. A significant difference was considered at *p* < 0.05.

## Results

### Hyperoxia Suppressed Lung HSP70 Expression, Whereas GGA Enhanced it

**Fig. 1:**
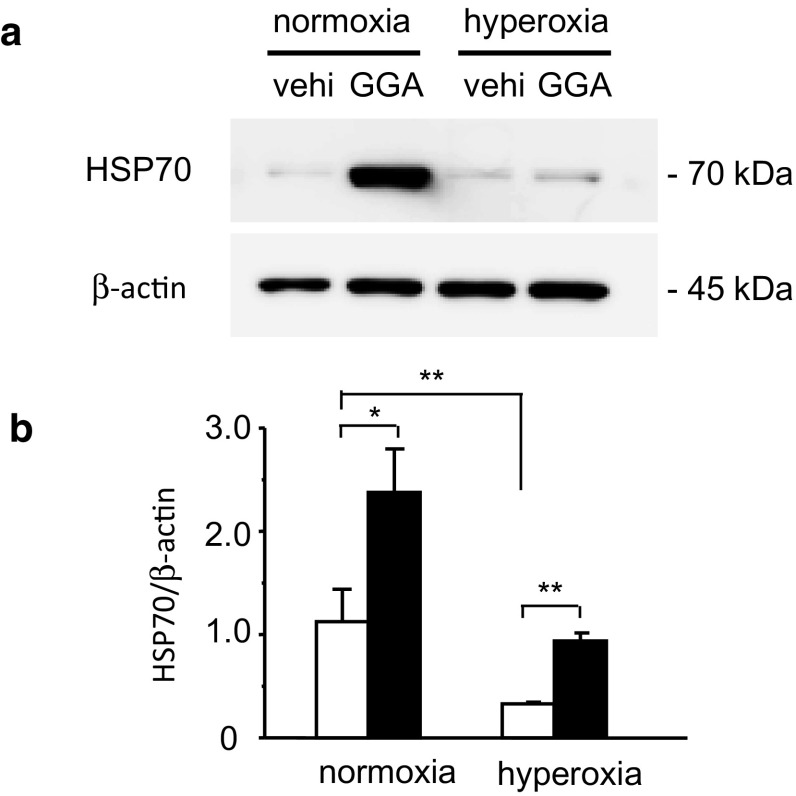
Geranylgeranylacetone (GGA)-induced expression of heat shock protein 70 (HSP70) in the lungs. **a** Representative immunoblotting data for HSP70 and β-actin expressions 24 h after the administration of GGA or vehicle alone (vehi) under normoxia or hyperoxia are shown. **b** Expression levels of HSP70 normalized by β-actin signal were calculated by densitometry and compared between the vehicle-treated group (*open bars*) and the 500 mg/kg GGA-treated group (*closed bars*). *vehi* vehicle administration. Data shown represent means ± the standard error of the mean (SEM) (normoxia/vehicle group: *n* = 8; normoxia/GGA group: *n* = 7; hyperoxia/vehicle group: *n* = 6; hyperoxia/GGA group: *n* = 7). Comparison between the indicated groups (*p < 0.05, **p < 0.01)

We first examined the effects of GGA administration and hyperoxia exposure on HSP70 expression in the lung. HSP70 protein expression was enhanced 24 h after in vivo GGA administration (Fig. 1). HSP70 expression levels in the lungs treated with 500 mg/kg GGA were more than two-fold higher than those treated with vehicle alone (2.4 ± 0.4 vs. 1.1 ± 0.3, *p* < 0.05). In contrast, 24 h of hyperoxia exposure suppressed HSP70 protein expression compared with normoxia (0.3 ± 0.0 vs. 1.1 ± 0.3, *p* < 0.05). GGA administration abrogated hyperoxia-induced suppression of HSP70 protein expression compared with vehicle administration (0.9 ± 0.1 vs. 0.3 ± 0.0, *p* < 0.01).

### GGA Improved Hyperoxia-Induced Failure to Thrive

**Fig. 2:**
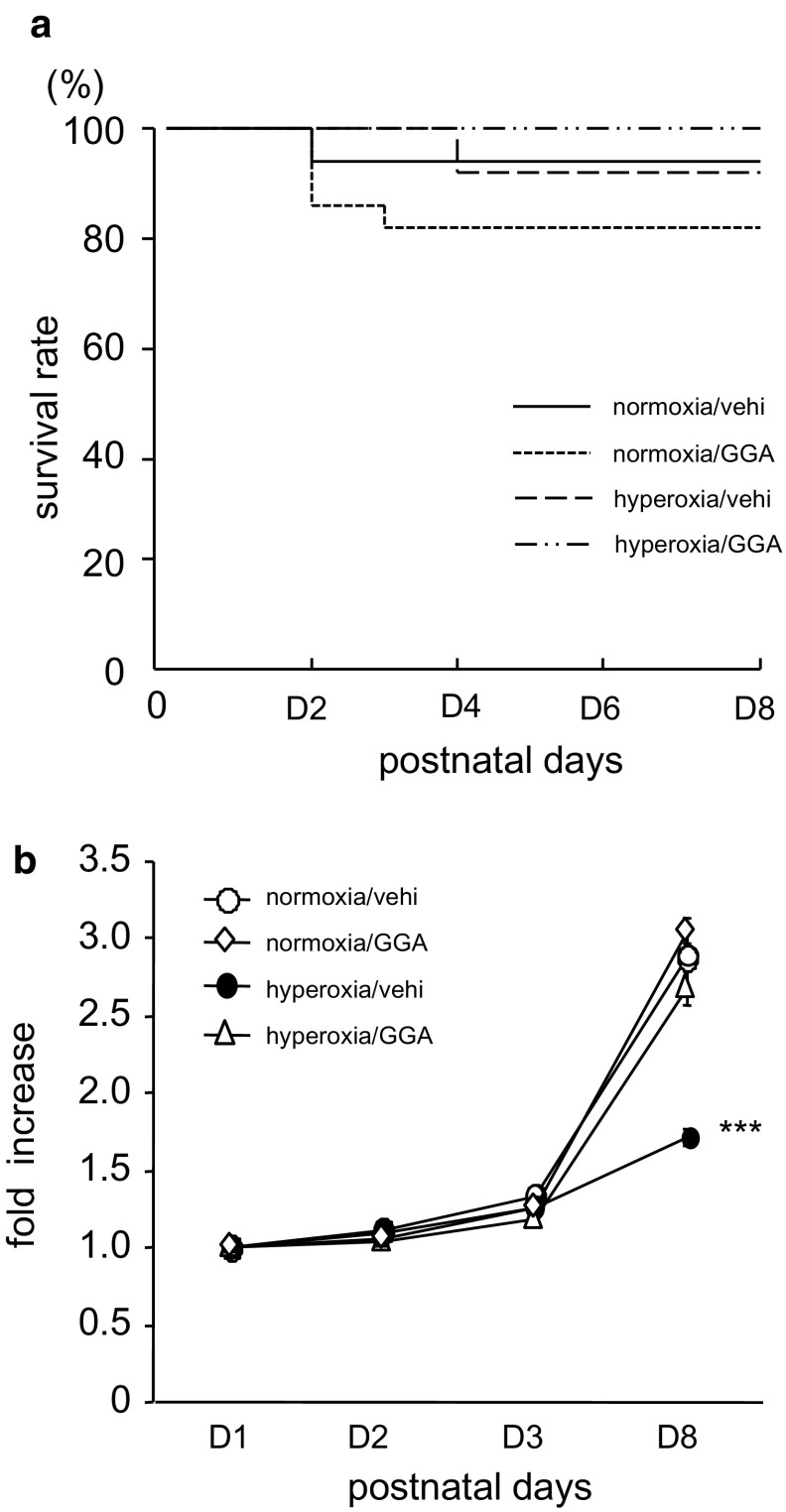
**a** Pup survival rate. The total number of pups treated postnatally was 15 in the normoxia/vehicle group, 22 in the normoxia/GGA group, 13 in the hyperoxia/vehicle group, and 15 in the hyperoxia/GGA group, respectively. The Kaplan–Meier plot shows that the survival rate of pups was not significantly different among groups. **b** GGA treatment prevented hyperoxia-induced failure to thrive. Data are expressed as rates of increase from body weight at 1 day of age. Data shown represent mean ± SEM (normoxia/vehicle group: *n* = 6; normoxia/GGA group: *n* = 6; hyperoxia/vehicle group: *n* = 7; hyperoxia/GGA group: *n* = 6). ***Comparison between the indicated group and normoxia/vehicle, normoxia/GGA, and hyperoxia/GGA groups (***p < 0.001)

The total number of pups treated postnatally was 15 in the normoxia/vehicle-treated group, 22 in the normoxia/GGA-treated group, 13 in the hyperoxia/vehicle-treated group, and 15 in the hyperoxia/GGA-treated group, and the survival rate in each group was 93.3, 81.8, 92.3, and 100%, respectively. Pup survival rate was not significantly different among groups (Fig. 2a). None of the total 12 pregnant mice swapped every 24 h between normoxia and hyperoxia groups died during the experimental period. Hyperoxia exposure significantly reduced body weight gain rate (Fig. 2b). At 8 days of age, the body weight of hyperoxia/vehicle-treated neonates was 1.7 ± 0.1 times the body weight at day 1, whereas in normoxia/vehicle-treated neonates it was 2.9 ± 0.2 times the body weight at day 1 (*p* < 0.001). Treatment with 500 mg/kg GGA did not affect the body weight gain of neonates kept in room air. Of note, GGA treatment improved the hyperoxia-induced reduction in body weight gain.

### GGA Restored Hyperoxia-Induced Hypo-Alveolarization

**Fig. 3:**
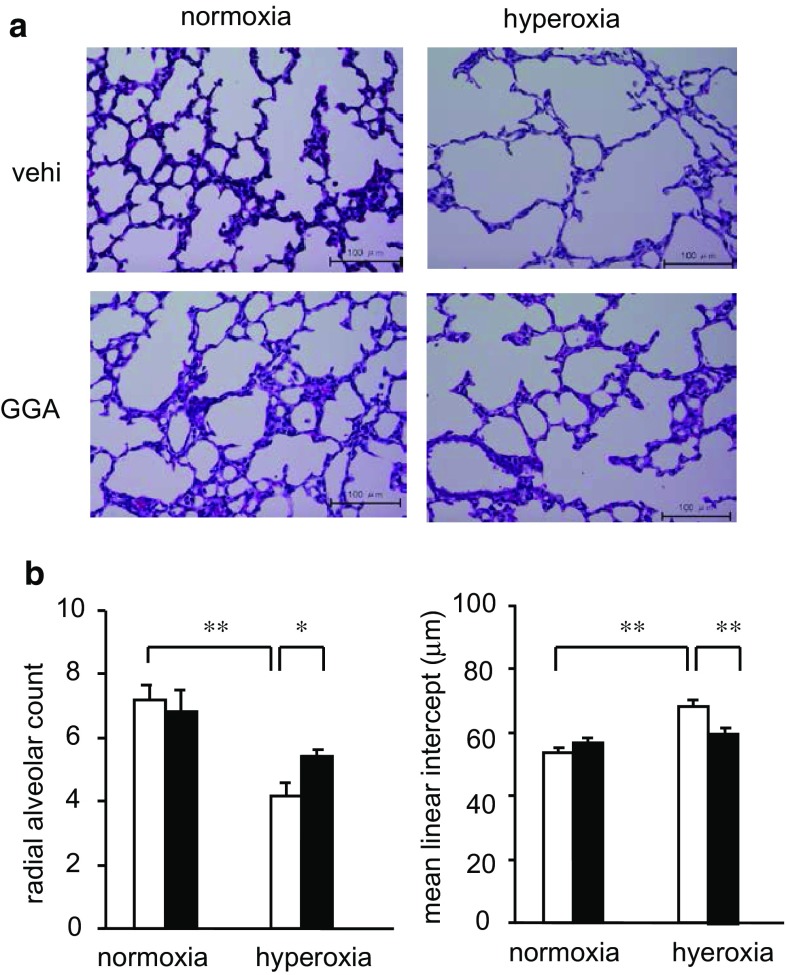
GGA treatment reverted histological changes induced by hyperoxia. **a** Histology of neonatal lungs at 8 days of age. All images at ×400 magnification. *Scale bar*: 100 μm. **b** Radial alveolar count (RAC) and mean linear intercept (MLI) were compared between the vehicle-treated group (*open bars*) and the 500 mg/kg GGA-treated group (*closed bars*). Data are the mean ± SEM (normoxia/vehicle group: *n* = 6; normoxia/GGA group: *n* = 6; hyperoxia/vehicle group: *n* = 7; hyperoxia/GGA group: *n* = 6). Comparison between the indicated groups (*p < 0.05, **p < 0.01)

As just 3 days of hyperoxia exposure led to the subsequent failure of neonates to thrive, we investigated whether the hyperoxia resulted in any observable sequelae in neonatal lungs. The lungs of neonates exposed to hyperoxia showed smaller RAC and larger MLI than those of neonates kept in normoxic conditions (RAC: 4.2 ± 0.4 vs. 7.2 ± 0.5, *p* < 0.001; MLI: 68.4 ± 1.9 vs. 53.7 ± 1.4 μm, *p* < 0.001) (Fig. 3). Thus, 3 days of hyperoxia exposure resulted in hypo-alveolarization or emphysematous changes with significantly decreased RAC and increased MLI. Treatment with 500 mg/kg GGA increased RAC and decreased MLI in the lungs of hyperoxia-exposed neonates, suggesting that GGA suppressed hyperoxia-induced lung injuries (RAC: 5.4 ± 0.2 vs. 4.2 ± 0.4, *p* < 0.05; MLI: 59.9 ± 1.6 vs. 68.4 ± 1.9 μm, *p* < 0.01).

### GGA Exhibited Cytoprotective Effects Against Hyperoxia-Induced Lung Injury

**Fig. 4:**
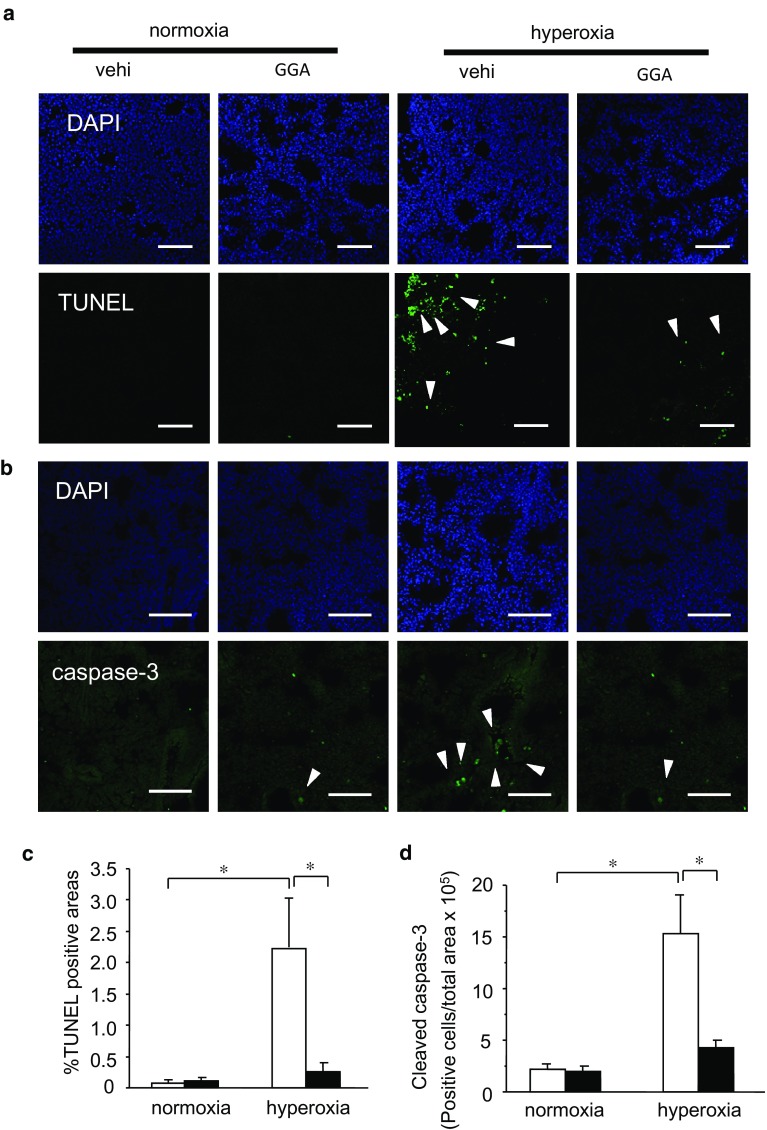
GGA treatment inhibited hyperoxia-induced apoptosis in lungs. Nucleus and apoptotic cells were identified via DAPI staining and the terminal deoxynucleotidyl transferase-mediated dUTP nick end labeling (TUNEL) method (**a**), and cleaved caspase-3 immunohistochemistry (**b**) in the hyperoxia-exposed neonatal lungs treated with 500 mg/kg GGA or vehicle alone. TUNEL-positive areas (**c**) and cleaved caspase-3-positive cells (**d**) expressed per the total area of the lung section of each slide were compared between the vehicle-treated group (*open bars*) and the GGA-treated group (*closed bars*) under normoxia or hyperoxia. *Arrows* indicate TUNEL-positive cells (**a**) and cleaved caspase-3-positive cells (**b**). All images at ×200 magnification. *Scale bar*: 100 μm. Data are the mean ± SEM (normoxia/vehicle group: *n* = 6; normoxia/GGA group: *n* = 6; hyperoxia/vehicle group: *n* = 7; hyperoxia/GGA group: *n* = 6). Comparison between the indicated groups (*p < 0.05)

To clarify the mechanisms involved in the structural changes observed in hyperoxia-exposed neonatal lungs, we examined apoptosis of pulmonary parenchymal cells via the TUNEL assay. Hyperoxia exposure for 3 days apparently increased the number of TUNEL-positive cells in neonatal lungs at 8 days of age (Fig. 4a, c). In conjunction with structural changes, GGA treatment dramatically inhibited the increase in TUNEL-positive cells associated with hyperoxia exposure, suggesting that GGA treatment prevented hyperoxia-induced apoptosis. We also assessed cleaved caspase-3, a marker of apoptosis. Hyperoxia-exposed mice exhibited more caspase-3-positive cells in lungs than did normoxia-exposed mice. GGA significantly suppressed the hyperoxia-induced increase in caspase-3-positive cells in lungs (Fig. 4b, d).

### Serum HSP70 Levels in Human Infants Decreased with Age

**Fig. 5:**
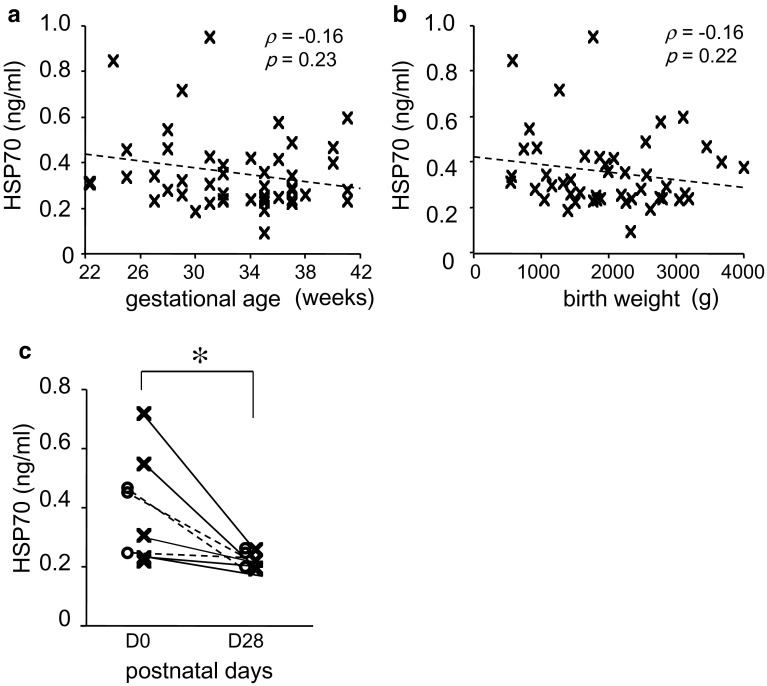
Correlations between serum HSP70 levels and gestational age **(a)** and birth weight **(b)**. Chronological changes in serum HSP70 levels in preterm human infants with or without BPD (*open circle* and *dashed line*, BPD, *n* = 4; *cross* and *solid line*, no-BPD, *n* = 8) **(c)**. Data are the median and range. *p < 0.05

Fifty-seven infants were enrolled in this study; median gestational age was 35 weeks (range 22–42 weeks), median birth weight was 1951 g (range 541–4089 g), median Apgar score at 1 min was 8 (range 0–9), and median Apgar score at 5 min was 9 (range 4–10). Twenty-eight infants (49.1%) were male, 25 (43.9%) received prenatal steroids, 17 (29.8%) received surfactant, 6 (10.5%) had chorioamnionitis, and 7 (12.3%) had BPD. Serum HSP70 levels between postnatal days 0 and 3 were not significantly correlated with gestational age, birth weight, or Apgar score. While serum HSP70 levels in infants administered surfactant at birth were significantly higher than those of non-administered infants (median 0.34 ng/ml, range 0.22–0.96 ng/ml vs. median 0.26 ng/ml, range 0.10–0.60 ng/ml; *p* < 0.05), there were no significant effects of sex, antenatal steroid use, chorioamnionitis, or the development of BPD on serum HSP70 levels at postnatal days 0–3. Notably, serum levels significantly decreased as the infants aged (median 0.33 ng/ml, range 0.22–0.72 ng/ml vs. median 0.20 ng/ml, range 0.18–0.37 ng/ml; *p* < 0.05). There were no statistically significant differences in the serum HSP70 levels at postnatal days 28–30 between the infants with and without BPD (Fig. 5).

## Discussion

We demonstrated that GGA administration induced HSP70, which may have exerted anti-apoptotic and cytoprotective effects against hyperoxia insults, resulting in improved hypo-alveolarization of neonatal lungs in a mouse BPD model. Although oxygen therapy is essential for saving the lives of immature infants, prolonged hyperoxia exposure may cause lung injury through the production of highly reactive and destructive oxygen radicals, resulting in BPD. In mouse models, hyperoxia-exposed neonatal lungs exhibit a severely simplified and enlarged alveolar structure, resembling the pathological changes observed in BPD [17]. Several experimental approaches using anti-oxidants, growth factors, or stem cells have been shown to prevent the development of BPD or restore normal alveolar structure. However, most of these approaches are not readily applicable to human neonates due to safety considerations.

GGA, an anti-ulcer drug, is a non-toxic HSP inducer [9]. Orally administered GGA at 600 mg/kg induces HSP70 expression in mouse lungs, starting at 8 h and reaching a maximum at 24 h [11]. In our pilot study, the expression levels of HSP70 in the lungs treated with a single dose of 250 mg/kg of GGA only tended to be higher than those treated with vehicle. Although 24 h of hyperoxia exposure suppressed HSP70 expression in neonatal lungs, Zhang et al. [8] reported that HSP70 mRNA expression is induced in adult mouse lung after ≥48 h of hyperoxia exposure. For these reasons, in the present study, we administered 500 mg/kg of GGA daily during 3 days of hyperoxia exposure. Zhang et al. [8] demonstrated that extracellular HSP70 transduces anti-apoptotic and cytoprotective signals through a toll-like receptor 4 and Trif-nuclear factor kappa B pathway, which induces Bcl-2 expression and inhibits caspase-3 activation. HSP70 expression in adult lungs after prolonged hyperoxia exposure may be due to an auto-regulatory protective mechanism against hyperoxia insults. Although apoptosis occurs in all stages of lung development, dysfunctional apoptotic activity may reduce alveolar number. In fact, in one study, prolonged ventilation-induced lung epithelial apoptosis in neonatal mice coincided with a significant reduction in alveolarization [18]. Thus, rapid induction of HSP70 by GGA could protect neonatal immature lung tissues from insults caused by reactive and destructive oxygen radicals. GGA administration may be a useful treatment for BPD.

HSP70 levels in the maternal and umbilical cord sera are up-regulated upon preterm delivery [19]. Gestational age, birth weight, and chorioamnionitis are known risk factors for the development of BPD. In the present study, none of these factors were directly associated with the neonatal HSP70 serum levels between postnatal days 0 and 3. HSP70 serum levels in human neonates are higher than those of normal adults, but gradually decrease from birth to 28–30 days of age. This transient increase in HSP70 may afford protection against excessive stress associated with birth. In the present study, oral GGA administration during hyperoxia exposure maintained the HSP70 levels and restored hypo-alveolarization of neonatal mice lungs. Full-term mice are born in the saccular stage of lung development, which corresponds to human lung development at 26–28 weeks of gestation. Mice lung development progresses to the alveolar stage at postnatal day 5, which corresponds to human lung development at 32–38 weeks of gestation [20]. Thus, our murine BPD model appears to be a useful model of therapeutic intervention with GGA in preterm human infants. If HSP70 expression was kept at high levels under mechanical ventilation and oxygen administration, the immature lung tissues may be protected from insults associated with BPD risk factors.

## Conclusion

Oral administration of GGA might be a useful and safe approach for the prevention of BPD caused by hyperoxia exposure. Further studies are required to elucidate the mechanisms underlying the development of alveolar structure in immature lungs, and the potential side effects of GGA.
